# Safety and feasibility of rotational atherectomy (RA) versus conventional stenting in patients with chronic total occlusion (CTO) lesions: a systematic review and meta-analysis

**DOI:** 10.1186/s12872-023-03673-2

**Published:** 2024-01-02

**Authors:** Ahmed Abdelaziz, Hanaa Elsayed, Aboalmagd Hamdaalah, Karim Atta, Ahmed Mechi, Hallas Kadhim, Aya Moustafa Aboutaleb, Ahmed Elaraby, Mohamed Hatem Ellabban, Fayed Mohamed Rzk, Mahmoud Eid, Hadeer Elsaeed AboElfarh, Rahma AbdElfattah Ibrahim, Emad Addin Zawaneh, Mahmoud Ezzat, Mohamed Abdelaziz, Abdelrahman H. Hafez, Shaimaa Fadel, Hazem S. Ghaith, Mustafa Suppah

**Affiliations:** 1 Medical Research group of Egypt (MRGE), Cairo, Egypt; 2 Faculty of Medicine, Al-Azhar University, Cairo, Egypt; 3 Faculty of Medicine, Zagazig University, Zagazig, Egypt; 4 Damietta Faculty of Medicine, Al-Azhar University, Damietta, Egypt; 5 Institute of Medicine, National Research Mordovia State University, Saransk, Russia; 6 Internal Medicine Department, Medicine College, University of Kufa, Najaf, Iraq; 7 College of Medicine, Al Muthanna university, Samawah, Iraq; 8 Faculty of Medicine, Menoufia University, Menoufia, Egypt; 9 Neurology Department, Faculty of Medicine, Mansoura University, Mansoura, Egypt; 10 Faculty of Medicine, Kafr Elsheikh University, Kafr Elsheikh, Egypt; 11 Faculty of medicine, Jordan university of science and technology, Irbid, Jordan; 12 Faculty of Medicine, Suez Canal University, Ismailia, Egypt; 13 Department of Cardiovascular Medicine, Mayo Clinic, 13400 E Shea Boulevard, Scottsdale, AZ 85259 USA

**Keywords:** RA, CTO lesions, Non-CTO lesions, PCI

## Abstract

**Background and aim:**

Interventional cardiologists face challenges in managing chronic total occlusion (CTO) lesions, with conflicting results when comparing rotational atherectomy (RA) to conventional PCI. This meta-analysis aims to provide a critical evaluation of the safety and feasibility of RA in CTO lesions.

**Methods:**

PubMed, Scopus, Web of Science, Ovid, and Cochrane central library until April 2023 were searched for relevant studies. MACE was our primary outcomes, other outcomes were all cause of death, cardiac death, MI, and TVR. Also, we reported angiographic outcomes as technical success, procedural success, and procedural complications in a random effect model. The pooled data was analyzed using odds ratio (OR) with its 95% CI using STATA 17 MP.

**Results:**

Seven studies comprising 5494 patients with a mean follow-up of 43.1 months were included in this meta-analysis. Our pooled analysis showed that RA was comparable to PCI to decrease the incidence of MACE (OR = 0.98, 95% CI [0.74 to 1.3], *p* = 0.9). Moreover, there was no significant difference between RA and conventional PCI in terms of other clinical or angiographic outcomes.

**Conclusion:**

Our study showed that RA had comparable clinical and angiographic outcomes as conventional PCI in CTO lesions, which offer interventional cardiologists an expanded perspective when addressing calcified lesions.

**PROSPERO registration:**

CRD42023417362.

**Supplementary Information:**

The online version contains supplementary material available at 10.1186/s12872-023-03673-2.

## Introduction

A chronic total occlusion (CTO) of coronary vessel is defined a complete blockage of the vessel, with no blood flow through the blocked segment according to the Thrombolysis in Myocardial Infarction (TIMI) grading system, and an estimated occlusion duration of ≥ 3 months [1–3]. CTOs are commonly seen in practice, accounting for approximately 20% of all patients referred for coronary angiography [4, 5]. Between 30 and 50% of patients with established coronary artery disease (CAD) have CTOs [5], and their prevalence is higher in patients who have undergone coronary artery bypass graft (CABG) surgery [4–6].

In general, percutaneous coronary intervention (PCI) for patients with CTO is a challenging procedure and requires not only patience during manipulation, but also requires experience with various instruments and interventional techniques [7, 8]. With the advent of hybrid treatment strategies, the recent success rate for treating CTO using conventional procedures has reached 60–92% when performed by skilled interventionists [9, 10]. However, heavily calcified stenoses present a challenge due to their refractory plaque burden and uneven lesion surface. This can be a contributing factor in the failure of stent delivery or insufficient stent expansion [11–14]. During CTO PCI, the most common retrograde CTO crossing technique is reverse controlled antegrade and retrograde subintimal tracking (reverse CART) [15]. When performing retrograde CTO-PCI in severely calcified lesions, the use of retrograde crossing techniques, in particular reverse CART, has been thought to carry a relatively high risk of dissection and perforation after rotational atherectomy (RA) in these lesions [16].

Rotational atherectomy has been considered to be the most promising intervention in the treatment of CTO lesions and might improve the procedure success rate and it is increasingly applied for the preparation of certain calcified CTOs [17, 18]. Rotational atherectomy is a process that involves cutting a portion of the obstructive atheroma to enable balloon dilatation, plaque fracture, stent delivery, and expansion [19, 20]. It is utilized for complete lesion preparation prior to stenting as well as device passage [21]. However, despite the potential for short-term results of CTO PCI, it has been observed that it might be consistent with a higher possibility of peri-procedural complications [22]. Moreover, the long-term clinical outcome of CTO lesions treated by RA-assisted PCI remains unclear.

At present, the optimal treatment for CTO lesions is still debatable due to conflicting results from the randomized controlled trials (RCTs) comparing RA to non-RA strategies in managing CTO lesions. To date, no systematic review and meta-analysis has compared the preparation of calcified coronary lesions with an atherectomy-based strategy to non-RA PCI in the treatment of CTO lesions. Therefore, we conducted a systematic review and meta-analysis to compare the outcomes and efficacy of these approaches.

## Methods

We followed the preferred reporting items for systematic reviews and meta-analysis (PRISMA) statement guidelines when performing this systematic review and meta-analysis [23]. The method was carried out in accordance with the Cochrane handbook of systematic reviews and meta-analysis of interventions (version 5.1.0).

### Eligibility criteria

We considered all relevant trials that reported RA as the interventional group and conventional PCI method as the comparable group in patients with chronic total occlusion and reported our primary and secondary outcomes of interest. We excluded irrelevant papers of animal studies, non-English studies, data of unpublished studies, or data from conference abstracts.

### Primary and secondary outcomes

The primary outcome of interest was the incidence of major adverse cardiac events (MACE) as a clinical outcome of interest. Other secondary outcomes were all cause of death, cardiac death, myocardial infarction (MI), and target vessel revascularization (TVR). Also, we reported angiographic outcomes as technical success, procedural success, and procedural complications. The definitions of these outcomes were based on the descriptions provided by the authors of the individual studies.

### Literature search

We performed a comprehensive literature search on PubMed, Scopus, Web of Science, and Cochrane Library, from inception until April 2023, using this search query: (“rotational atherectomy” OR RA) AND (“coronary artery disease” OR “chronic total occlusion” OR CTO OR “CTO lesions” OR “CTO-PCI”) AND (“non-RA” OR “without RA” OR “stenting without RA” OR “conventional PCI” OR “standard PCI”) AND (“percutaneous coronary intervention” OR PCI OR “drug-eluting stent*” OR DES). All duplicates were removed by EndNote and manual backward citation analysis was done for all the references of the included studies.

### Screening of the literature search results

The literature search results were screened in a two-step process. Initially, the titles and abstracts of all articles were assessed for eligibility. Subsequently, full-text screening was conducted for the studies that met the eligibility criteria.

### Data extraction

Data from the included studies was extracted and recorded in a standardized data extraction sheet. The extracted data encompassed four main categories: (1) Characteristics of the included studies, (2) Characteristics of the study population, (3) Risk of bias domains, and (4) Outcome measures, which included MACE, All-Cause Death, TVR, MI, stent thrombosis, and cardiac death.

### Synthesis of results

For outcomes that involved dichotomous data, the frequency of events and the total number of patients in each group were combined to calculate the odds ratio (OR) with its 95% confidence interval (CI) using the DerSimonian-Laird random-effect model, moreover, a *P* value less than 0.05 was considered as a significant result. In cases where studies reported data at multiple time points, the last endpoint was considered for the primary analysis. All statistical analyses were conducted using StataMP version 17 for Mac.

### Assessment of Heterogeneity

Statistical heterogeneity among studies was evaluated by the Chi-square test (Cochrane Q test). Next, the chi-square statistic, Cochrane Q, was used to calculate the I-squared according to the equation: I2=$\left(\frac{Q-df}{Q}\right)\, x\, 100\%$. A Chi-square *P* value less than 0.1 was considered as significant heterogeneity. I-square values ≥ 50% were considered to indicate high heterogeneity. In scenarios of significant heterogeneity, we employed the leave-one-out sensitivity analysis model to address the reported heterogeneity. Moreover, we used the Galbraith plot to detect any heterogeneity across studies.

### Quality assessment

To assess the quality of the clinical trials included in our analysis, we utilized the Cochrane Risk of Bias 2 (ROB-2) tool for randomized controlled trials (RCTs) [24]. This tool evaluates the risk of bias in five domains, namely, selection bias, performance bias, detection bias, attrition bias, and reporting bias. The authors’ decisions were categorized as ‘High risk of bias’, ‘Some concerns’, or ‘Low risk of bias’. Observational studies were assessed using the Newcastle–Ottawa Scale (NOS) [25] which involves three domains (selection, comparability, and outcome).

To investigate publication bias across studies, we employed the DOI plot model to analyze the relationship between effect size and standard error [26]. We assessed evidence of publication bias using Egger’s regression test [27].

## Results

### Literature search

**Fig. 1:**
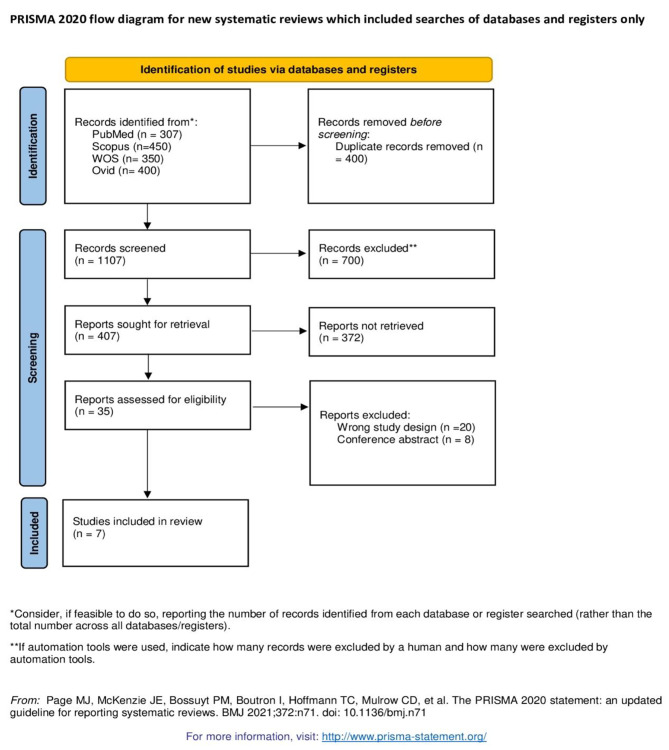
PRISMA flow diagram

Our electronic search on four databases yielded 1507 articles of which seven studies were finally included in this meta-analysis after title, abstract, and full text screening. The flow chart of included studies is illustrated in PRISMA flow diagram, as shown in Fig. 1.

### Characteristics of included studies

Our meta-analysis included seven studies with a total of 5494 patients. Of the included studies, 5 were observational studies [8, 28]– [31], and only two were randomized controlled trials [13, 14]. The baseline summary and characteristics of all included studies is summarized in Table 1.

**Table 1:** Summary and baseline of all included studies

Characteristics of All included Studies																													
Author, year	Type of Study	Country	Patients (n) (comp/control)	Age (mean) (comp/control)	male, n (comp/control)	mean follow up	stent length, mean (comp/control)	Number of stents, mean (comp/control)	Reference vessel diameter, mean (comp/control)	Lesion length, mean (comp/control)	Diameter stenosis, mean (comp/control)	Medical conditions, n (comp/control)								Target vessel, n (comp/control)				Procedural Characteristics, n (comp/control)					
												Previous MI	Previous CABG	Previous PCI	DM	HTN	Dyslipidaemia	Current smoker	PAD	LM	LCX	RCA	LAD	Ostial location	Bifurcation	Moderate/severe tortuosity	Severe calcification	B2/C lesion	7 F guiding catheter
Wang 2022	Retrospective study	China	16/313	(60.87/59.70)	(16/284)	30.22 month	(105.5/90.69)	(3.14/2.50)	NA	NA	NA	(4/79)	(0/15)	(10/206)	(8/95)	(14/184)	(5/89)	(3/70)	NA	(0/1)	(0/7)	(12/185)	(4/120)	NA	NA	(14/184)	(16/157)	NA	NA
Pagnotta 2010	Retrospective study	Italy	45/603	(70/63)	(29/506)	NA	NA	NA	(2.75/ 2.79)	(26/22)	NA	(24/341)	(18/87)	NA	(20/250)	(34/512)	NA	(13/126)	NA	NA	(8/134)	(18/241)	(13/158)	(0/56)	NA	NA	(14/141)	NA	NA
Abdel-Wahab 2013	randomized active-controlled superiority trial	Germany	120/120	70.5/71.8	86/96	75.3 months	27.7/25.2	1./1.3	3.1/3.1	20.6 /18.5	81.5/80	38/29	9/15	44/39	33/32	106/95	91/87	24/16	NR	3/2	7/22	35/41	101/111	27/31	72/82	67/83	65/86	137/152	122/50
Abdel-Wahab 2018	randomized trial	Germany	100/100	75/74.8	75/77	77.1 months	35.41/35.63	1.71/1.52	3.31/3.25	30.07/29.81	83.54/83.02	22/21	13/6	55/47	34/33	93/93	69/68	9/15	NR	20/15	16/16	40/32	61/78	35/40	61/55	44/49	100/104	129/137	111/130
Azzalini 2017	Retrospective study	Spain, Canada, Italy	35/968	68.9/64.6	31/841	2 years	72.8/68.4	2/2.2	NR	NR	NR	NR	NR	NR	19/351	27/711	32/765	9/205	NR	NR	3/180	17/509	15/275	NR	NR	NR	30/413	NR	NR
Huang 2018	Retrospective study	Taiwan	26/259	71.1/66.4	23/215	3.4 years	40.7/34.2	NR	NR	NR	NR	NR	NR	NR	13/158	19/223	NR	4/11	NR	1/2	1/10	14/137	11/110	NR	NR	NR	NR	NR	NR
Ayoub 2023	Retrospective study	Germany	193/2596	70.33/65.73	156/2143	1 year	50.55/54.18	2.19/1.73	NR	NR	NR	66/995	65/417	NR	84/775	178/2229	178/2333	21/519	NR	NR	37/554	NR	40/699	14/174	NR	46/538	166/942	NR	117/889

### Risk of bias assessment

The Five observational studies were truly representative of the patients included. The non-exposed group was selected from the same community and the ascertainment of exposure was confirmed by secure records. Furthermore, the two groups included in all the studies were comparable. They also showed adequate periods of follow-up except for Pagnotta 2010. However, the overall quality of all the studies is good, as shown in Table 2.

**Table 2:** NOS scale for observational studies

Cohort studies															
Baseline							Selection			Comparability	Outcome				Quality Score
Study Title	First Author	Year	Study Design (Prospective or retrospective)	mean follow up	Sample (n) (comp/control)	Age at baseline mean (Year) (comp/control)	Representativeness of the exposed cohort	Selection of the non-exposed cohort	Ascertainment of exposure	Demonstration that outcome of interest was not present at start of study	Comparability of cohorts on the basis of the design or analysis	Assessment of outcome	Was follow-up long enough for outcomes to occur	Adequacy of follow up of cohorts	
Ayoub 2023	Mohamed Ayoub	2023	Retrospective study	1 year	(193/2596)	(70.33/65.73)	*	*	*	*	**	*	*	*	Good
Wang 2022	Jing Wang	2022	Retrospective study	30.22 months	(16/313)	(60.87/59.70)	*	*	*	*	*	*	*	*	good
Pagnotta 2010	Paolo Pagnotta	2010	Retrospective study	Not applicable	(45/603)	(70/63)	*	*	*	*	*	*	*	*	good
Azzalini 2017	Lorenzo Azzalini	2017	Retrospective study	2 years	35/968	68.9/64.6	*	*	*	*	*	*	*	*	good
Huang2018	Wei-Chieh Huang	2018	Retrospective study	3.4 years	26/259	71.1/66.4	*	*	*	*	*	*	*	*	good

**Fig. 2:**
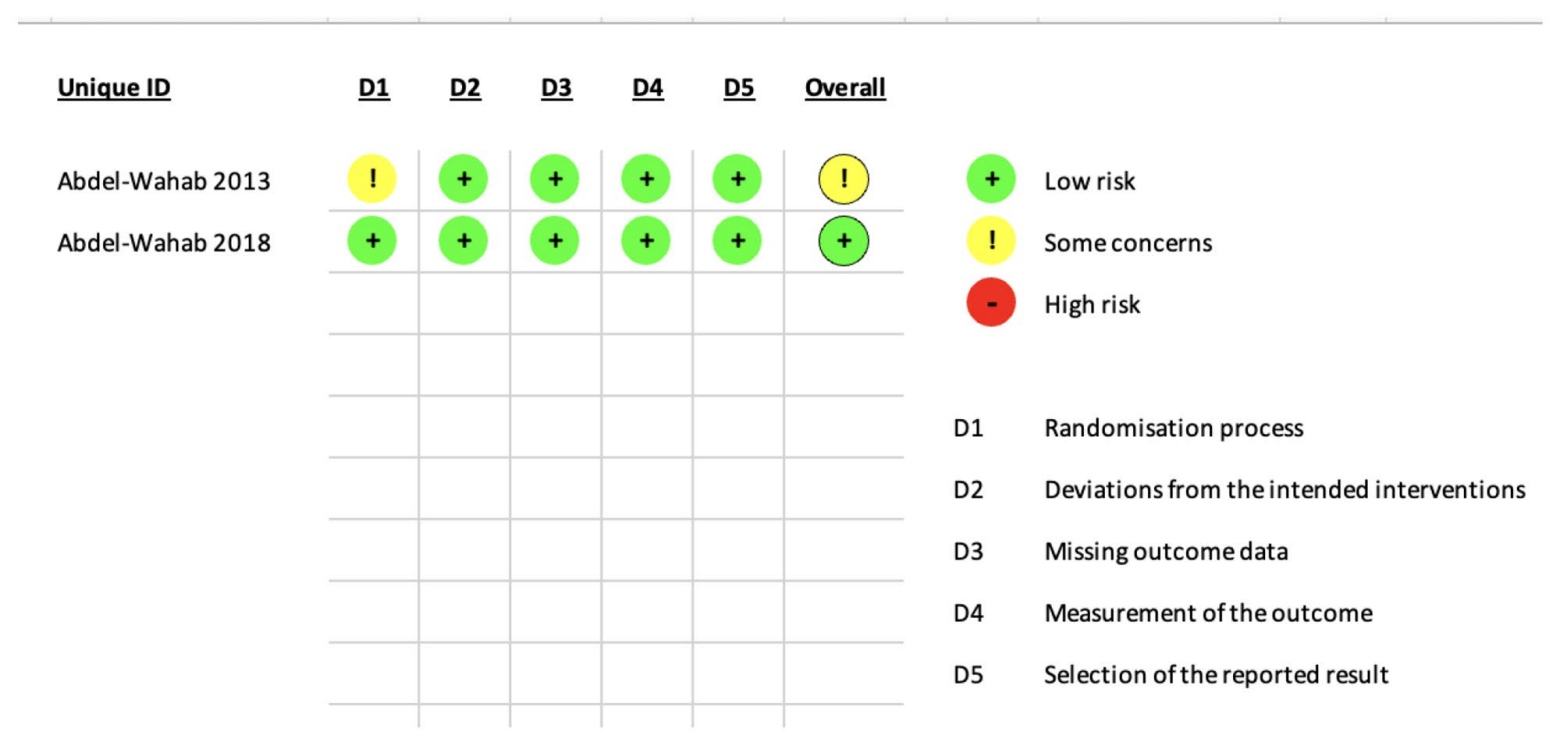
Risk of bias assessment tool-2 (ROB-2) for RCTs

The two RCTs Abdel-Wahab 2013 and Abdel-Wahab 2018 showed low risk of bias in almost all the five domains of Rob2. However, Abdel-Wahab 2013 showed protocol deviation in 3 patients. They demonstrated adequate randomization process, allocation concealment, appropriate analysis, appropriate outcome measurement, and no baseline difference between the two groups, as shown in Fig. 2.

### Clinical outcomes

#### MACE

**Fig. 3:**
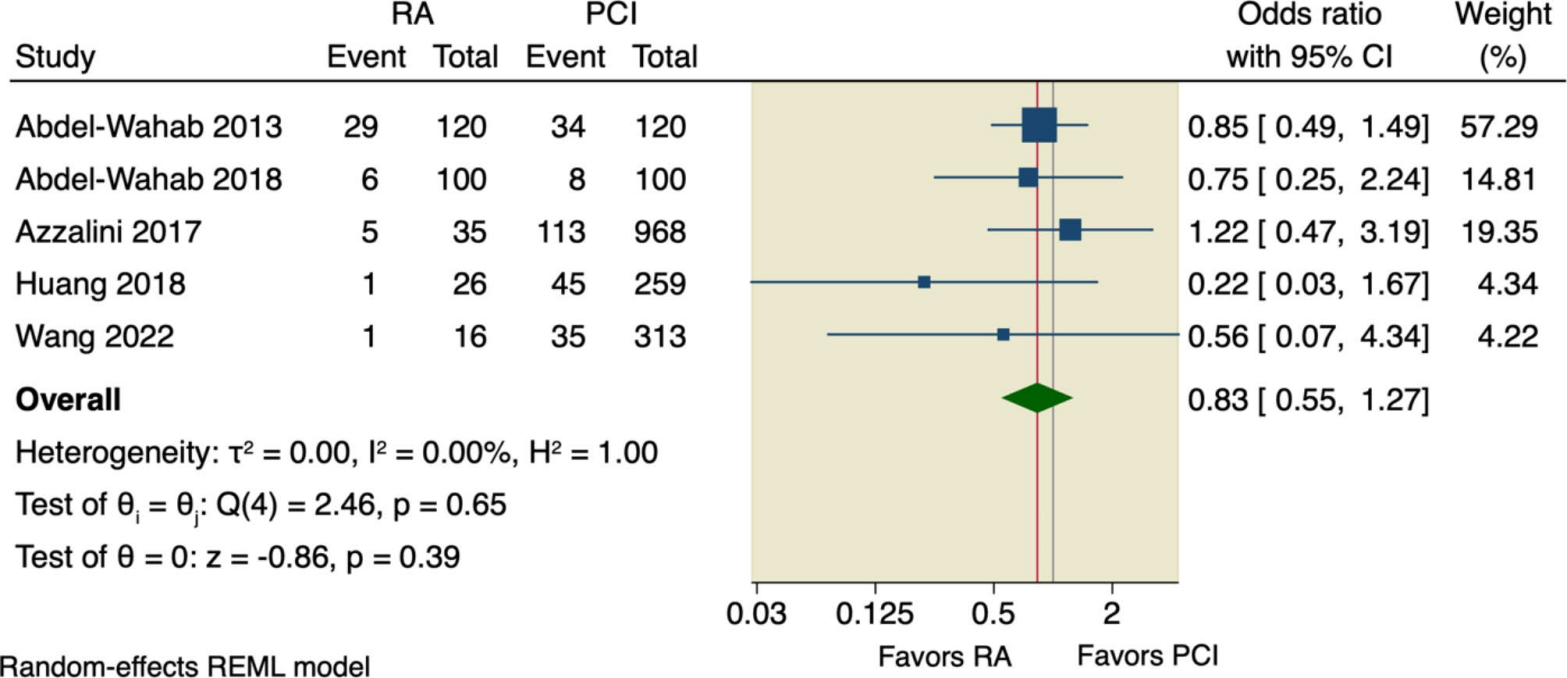
Forest plot of MACE. RA: rotational atherectomy, PCI: percutaneous coronary intervention, CI: confidence interval

Six studies assessed our primary outcome, MACE, with an incidence rate of 15.91% (78 of 490) in RA group compared to 15.36% (669 of 4356) in the conventional PCI group. The pooled OR did not detect any significant difference between the two studied groups regarding MACE (OR = 0.98, 95% CI [0.74 to 1.3], *p* = 0.9); the pooled studies were homogenous (I^2^ = 0.00%, *p* = 0.62), as shown in Fig. 3.

**Fig. 4:**
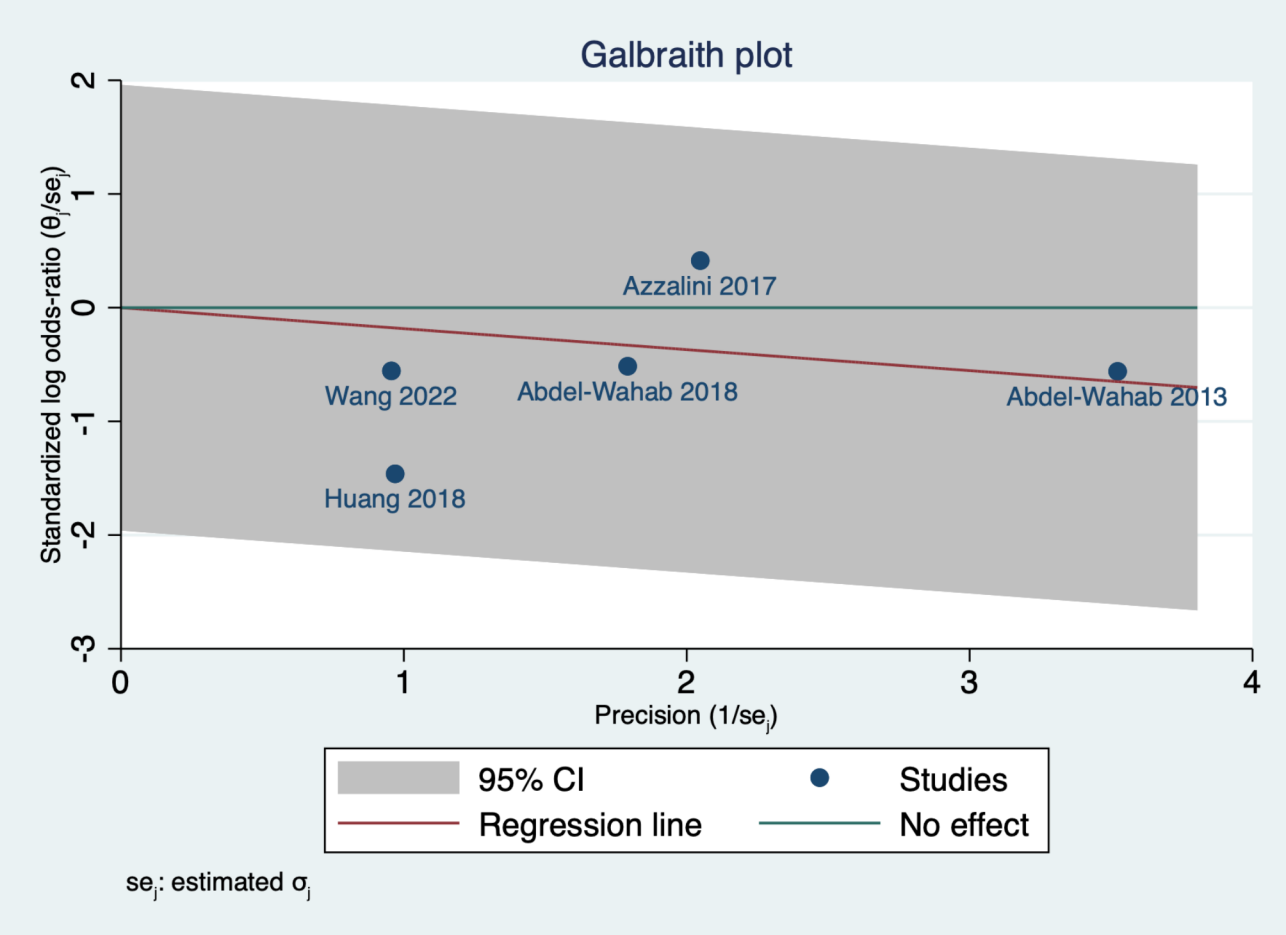
Galbraith plot assessing heterogeneity across studies assessed MACE

We assessed the statistical heterogeneity using Galbraith plot, and by inspection, all studies were within the 95% CI of the precision area, indicating that there no heterogeneity across studies, as shown in Fig. 4.

**Fig. 5:**
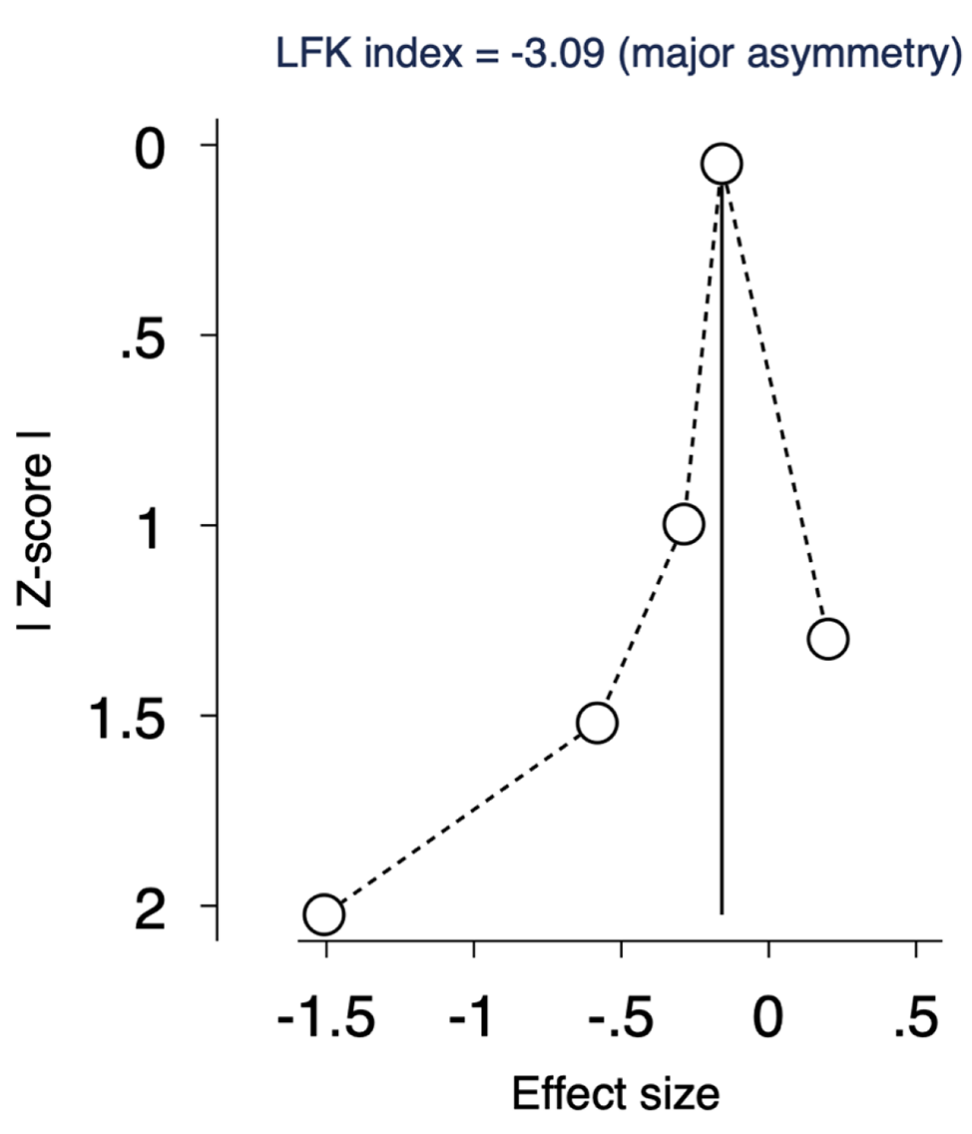
DOI plot assessing publication bias of MACE. LFK= -3.09, indicating major asymmetry

We used DOI plot to detect possible publication bias, and by inspection, there was a major asymmetry with a LFK of -4.7, indicating possible publication bias and further studies are needed to achieve stability, as shown in Fig. 5.

#### Secondary outcomes

**Fig. 6:**
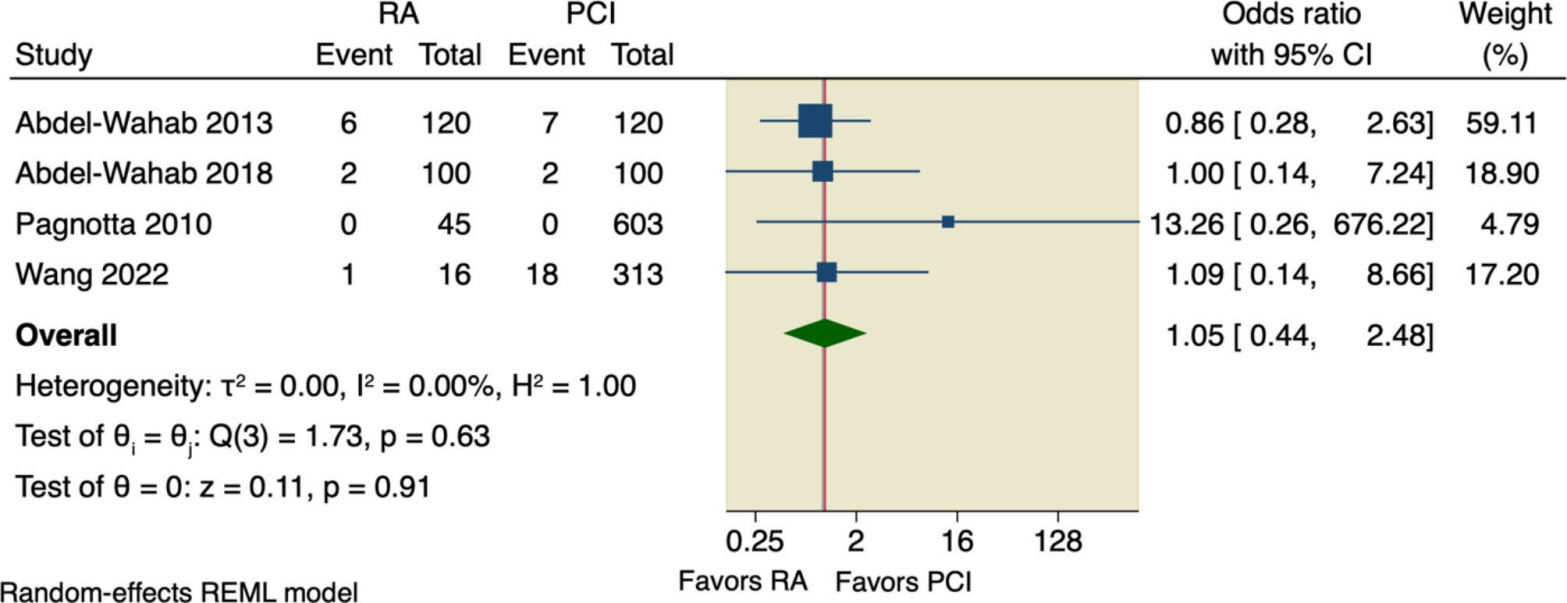
Forest plot of all-cause death. CI: Confidence interval, RA: rotational atherectomy, PCI: percutaneous coronary intervention

**Fig. 7:**
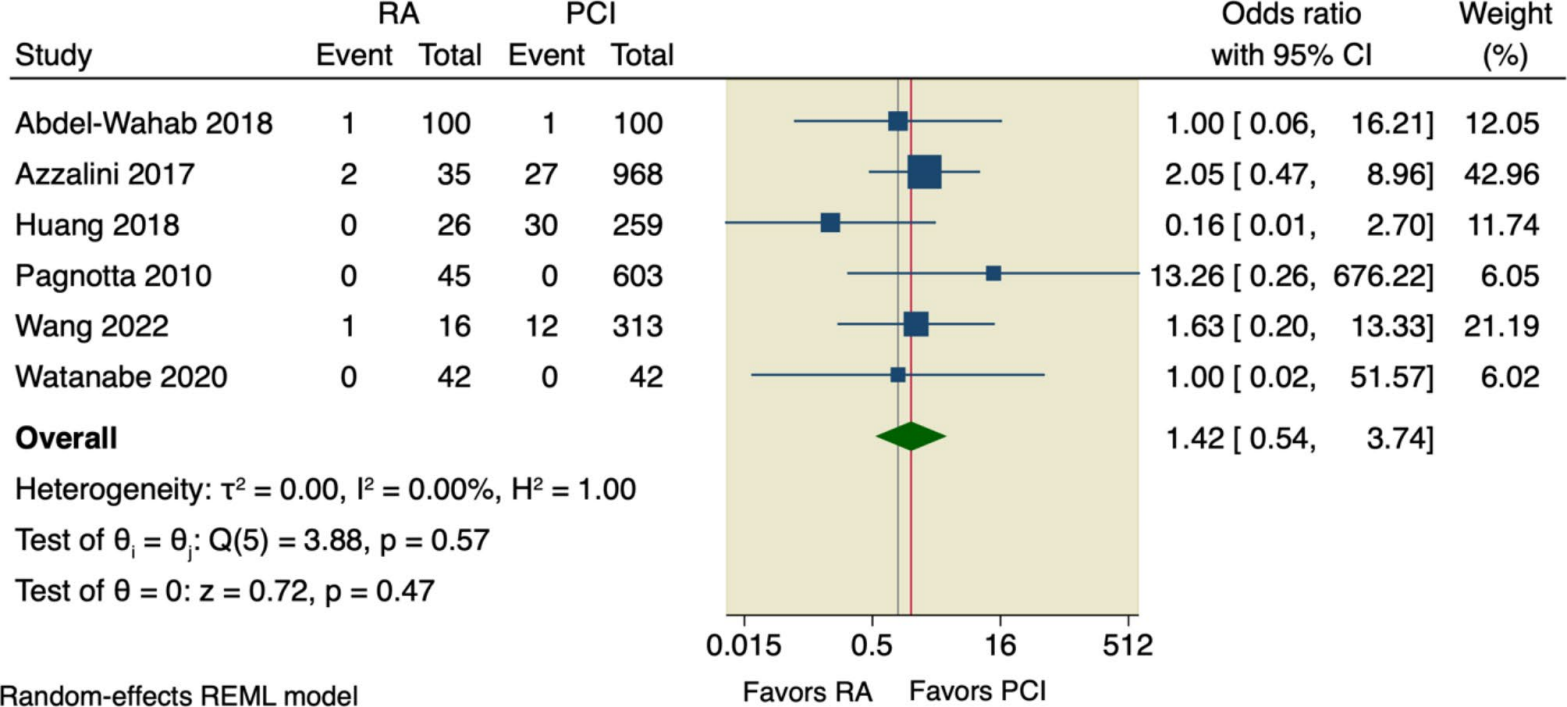
Forest plot of cardiac death. CI: Confidence interval, RA: rotational atherectomy, PCI: percutaneous coronary intervention

Our pooled analysis showed no significant difference between RA and conventional PCI according to all-cause death (OR = 1.34, 95% CI [0.8 to 2.25], *p* = 0.26), or cardiac death (OR = 1.46, 95% CI [0.54 to 3.95], *p* = 0.46); the pooled studies were homogenous with the following values, respectively (I^2^ = 0.00%, *p* = 0.69; and I^2^ = 0.00%, *p* = 0.43), as shown in Figs. 6 and 7.

Sensitivity analysis was done by excluding Pagnotta et al., in which the pooled studies did not favor RA over conventional PCI regarding either all-cause death (OR = 1.29, 95% CI [0.77 to 2.17], *p* = 0.33) or cardiac death (OR = 1.25, 95% CI [0.45 to 3.51], *p* = 0.67), as shown in Supplementary Figs. 1, 2.

**Fig. 8:**
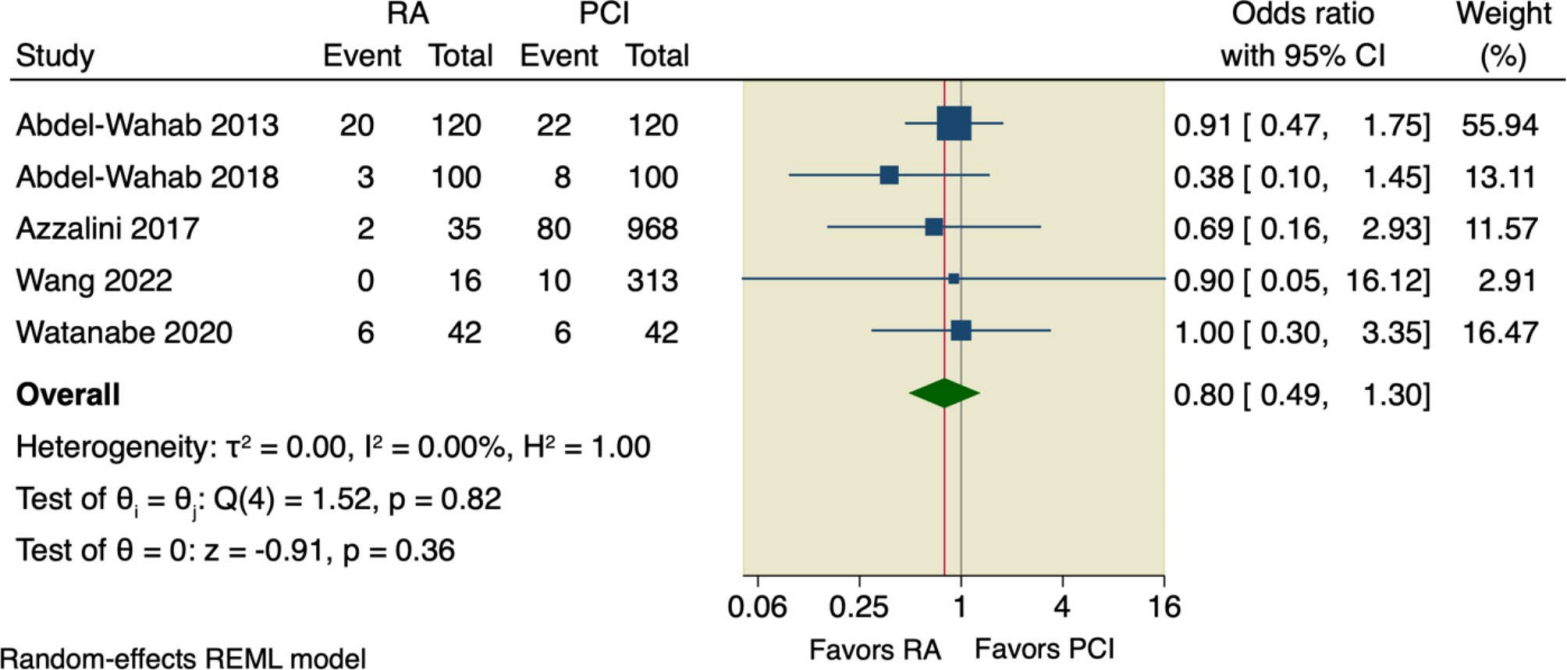
Forest plot of TVR. CI: Confidence interval, RA: rotational atherectomy, PCI: percutaneous coronary intervention

**Fig. 9:**
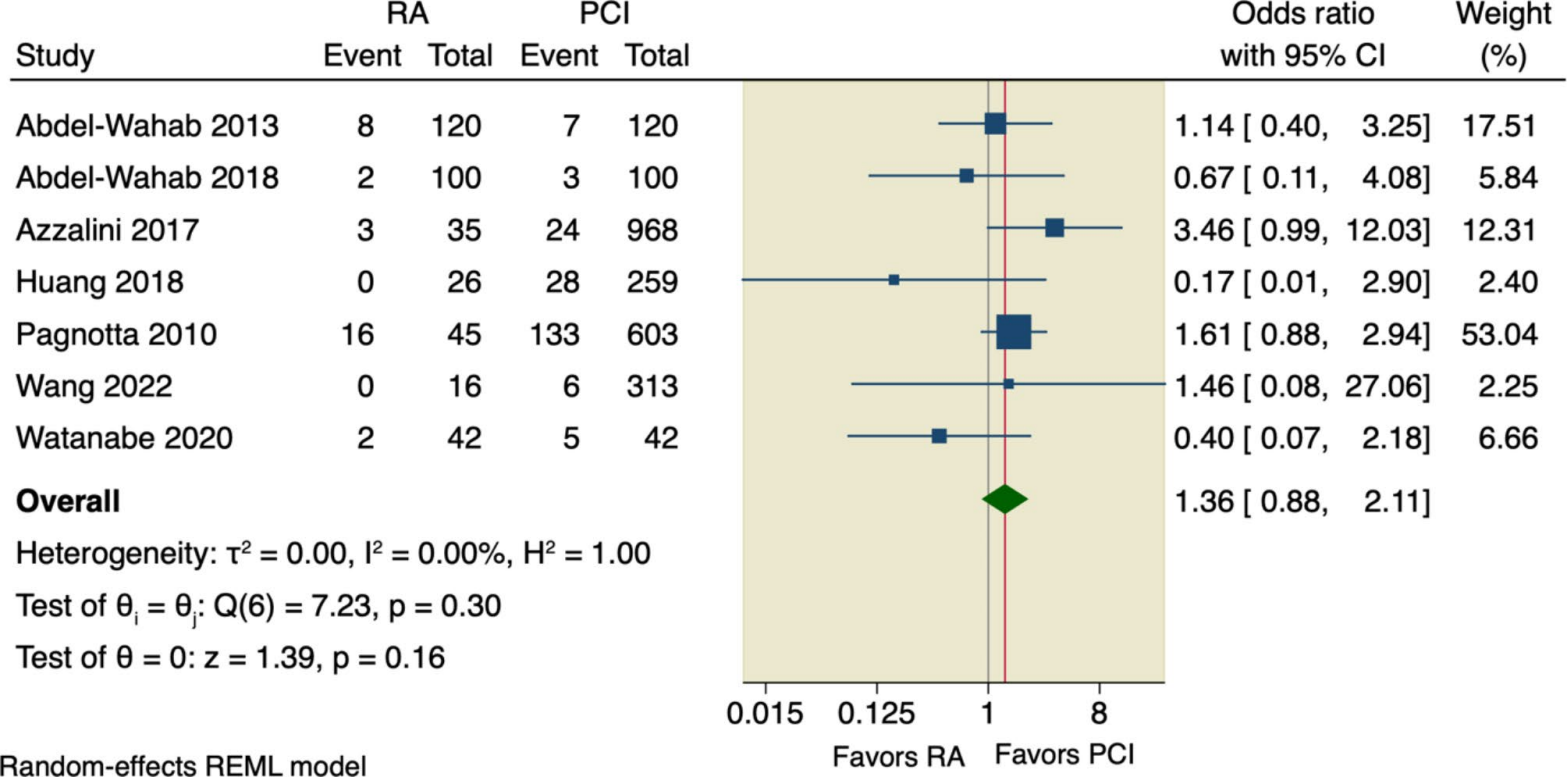
Forest plot of MI. CI: Confidence interval, RA: rotational atherectomy, PCI: percutaneous coronary intervention

Moreover, our pooled analysis showed no superior effect of RA compared to conventional PCI regarding the incidence of TVR (OR = 1.01, 95% CI [0.74 to 1.36], *p* = 0.97), or incidence of MI (OR = 1.42, 95% CI [0.92 to 2.18], *p* = 0.11); the pooled studies were homogenous with the following values, respectively (I^2^ = 0.00%, *p* = 0.58; and I^2^ = 0.00%, *p* = 0.47), as shown in Figs. 8 and 9.

### Angiographic outcomes

Four studies assessed technical success in which the pooled analysis did not favor RA over conventional PCI (OR = 1.06, 95% CI [0.89 to 1.25], *p* = 0.53); the pooled studies were homogenous (I^2^ = 0.00%, *p* = 0.87), as shown Supplementary Fig. 3. Regarding procedural success, the pooled analysis of six studies showed no superior effect of RA over conventional PCI (OR = 1.08, 95% CI [0.94 to 1.24], *p* = 0.29); the pooled results were homogenous (I^2^ = 0.00%, *p* = 0.98), as shown in Supplementary Fig. 4.

The incidence rate of procedural complications was 5.05% (15 of 297) in the RA group compared to 5% (88 of 1760) in the conventional PCI group. The pooled OR did not show any superiority of RA over conventional PCI (OR = 1.72, 95% CI [0.64 to 4.61], *p* = 0.28); the pooled studies were homogenous (I^2^ = 18.04%, *p* = 0.11), as shown in Supplementary Fig. 5.

A sensitivity analysis model was performed, and upon excluding Huang et al., the pooled analysis, yet, did not detect any difference between the two studied groups (OR = 1.34, 95% CI [0.55 to 3.28], *p* = 0.52), as shown Supplementary Fig. 6.

## Discussion

We conducted this meta-analysis to evaluate the safety and feasibility of RA in patients with CTO lesions compared to conventional CTO PCI. Our findings revealed that there was no significant difference between the two procedures in clinical outcomes, including MACE, all-cause death, cardiac death, incidence of TVR, and the incidence of MI. Similarly, we found no significant difference in angiographic outcomes, including technical success, procedural success, and complications.

Revascularization of CTO lesions can enhance long-term survival rates and quality of life, especially in patients with CTO in the left anterior descending territory and extensive ischemic areas [32]. Multiple studies have demonstrated the clinical benefits of successful canalization of CTO lesions in improving anginal pain, survival rate, and left ventricular systolic function [33, 34]. Given the numerous advantages of revascularization, it is essential to properly recanalize resistant CTO lesions.

CTO interventions represent around 25% of patients undergoing coronary angiography, and many interventional cardiologists are committed and enthusiastic about performing CTO PCIs. With well-trained physicians and technical advancements, the success rate for CTO PCIs has increased to over 80% after 15 years of follow-up. However, the mortality rate within 30 days of CTO PCI was 1.3%, and perforation occurred in 4.8% of cases [35, 36]. Another study with a 20-year follow-up found that successful CTO-PCI approaches were associated with a 10-year survival benefit [37].

Despite improvements in PCI technology and techniques, some lesions remain uncrossable or undilatable by balloon [38]. According to a previous multicenter CTO PCI registry, 9% of all lesions are considered uncrossable and are characterized by moderate to severe coronary calcification, moderate to severe coronary tortuosity, and higher J-CTO scores. Additionally, 12% of CTO lesions are undilatable, with most of them associated with a history of CAD, heart failure, diabetes, and higher J-CTO scores [39]. Uncrossable CTO lesions are associated with a lower success rate and more complications. Prolonged procedures also increase the amount of fluoroscopy time required [40].

The uncrossable CTO lesions can be successfully managed by special approaches such as deep seating of the guiding catheter, anchoring balloon technique, buddy wire technique, manual rotation of the Tornus catheter, and child-in-mother guiding catheters [41, 42]. Additionally, higher ballooning pressure, excimer laser, and RA showed a high success rate [38].

One of the most effective managements for resistant CTOs and heavy calcific lesions is RA [43]. Practice guidelines recommend using RA to prepare highly fibrous or calcified lesions that cannot be properly crossed by a balloon or dilated before stenting [36]. Fang et al. conducted a study to compare the feasibility and safety of RA versus a penetrating catheter (Tornus catheter) for treating heavy calcific lesions and it was found that RA was superior to the Tornus catheter in terms of in-hospital and 30-day follow-up outcomes. RA was associated with a higher device and angiographic success rate and a shorter procedural duration. However, both procedures showed similar results regarding major and minor complications [44]. Another study by Pagnotta et al. reported that the RA was superior to the penetrating catheter (Tornus catheter) with a predicted success rate of over 45%. In addition, the RA with a bail-out approach can increase the predicted success rate to 91% in CTO patients [45].

Previous randomized controlled trials reported that there were no significant differences between CTO-PCI and RA, which is in line with our results [13, 14, 46, 47]. The randomized ROTAXUS trial assessed the effect of the RA technique on heavily calcified lesions and fixation of a drug-eluting stent (DES), and they found that RA was not superior to other PCI procedures in terms of in-hospital outcomes and after 9 months of follow-up [13]. At 2-year clinical follow-up, there was no difference in MACE rates between patients with complicated calcified lesions undergoing RA and those undergoing standard PCI before DES implantation. However, the rate of MACE increased to one-third of total patients, which may be associated with the time-dependent occurrence of MACE [46].

The Randomized PREPARE-CALC trial showed similar rates of in-hospital and 9-month lumen loss when modified balloons or RA were used with a heavily calcific lesion [14]. Recently, a post hoc analysis of this trial showed that RA-based lesion preparation had a higher success rate than the modified balloon strategy-based lesion preparation in heavy calcific lesions in the left anterior descending artery. The major cause of this discrepancy was a greater rate of crossover and stent failure in the modified balloon group [47].

The ARTIST trial evaluated the efficacy and safety of RA followed by balloon angioplasty with balloon percutaneous transluminal coronary angioplasty, and no significant difference was found in the short-term success rate compared to other procedures. However, RA was associated with minimal luminal diameter after 6 months, considering that the operators used low-pressure inflations [48].

The wide range of atherectomy application rates during CTO PCI, ranging from 3.5 to 9%, is most likely due to variations in equipment accessibility and operator expertise [8, 31]. A previous retrospective study conducted in Europe and Russia on 3540 patients found that the currently used RA procedure, which is mostly utilized for complex lesions, has similar technical success rate compared to CTO-PCI, but carries a higher risk of donor vascular damage and tamponade, requiring pericardiocentesis. Additionally, after excluding failed crossing instances and those where the successful crossing technique was unclear. However, procedural success (94% vs. 96%, *p* = 0.358) and MACE (5% vs. 3%, *p* = 0.195) rates were similar [49]. Another study reported the similarity in success rate and MACE between both procedures in-hospital and after 1 year. Nevertheless, this benefit was lost when the multivariate Cox regression analysis was adjusted for confounding factors (HR 1.25, 95% CI, 0.33–1.94, *p* = 0.242) [8].

A previous study assessed the risk of RA and found that RA was associated with more dissections; however, there were no significant differences between CTO and non-CTO groups [50]. Additionally, the CTO-PCI has a higher rate of dissection, especially with the retrograde method compared with the antegrade method [51].

Moreover, a recent meta-analysis addressed the clinical outcomes of PCI in CTO lesions reported that PCI in CTO lesions was associated with higher odds of vessel perforations and cardiac tamponade during in-hospital stay, while high rates of MACE was noted during long-term outcomes highlighting the challenges and adverse endpoints related to PCI in CTO lesions [52].

In terms of clinical implications, our findings suggest that RA is an important procedure for managing uncrossable or undilatable lesions with a high success rate and less fluoroscopy time. However, CTO-PCI has comparable success rates and complications. Therefore, while RA may not be the preferred first choice for CTO lesions, it remains a practical and viable alternative for difficult-to-treat lesions.

The study has several strengths. It is the first meta-analysis to compare the effectiveness of RA to CTO-PCI in heavy and complex CTO lesions, and we obtained consistent results by combining data from studies with similar characteristics. Our findings have important clinical implications for the management of CTO lesions. However, the study also has some limitations. Firstly, most of the included studies were observational, which may have introduced bias. Secondly, the number of included studies was relatively small, and there was evidence of publication bias. Therefore, we suggest that more prospective clinical trials with larger sample sizes are needed to evaluate the short-term and long-term outcomes associated with RA and CTO-PCI, as well as to compare the different procedures within CTO-PCI and RA.

In conclusion, our results demonstrate that patients who underwent RA and CTO-PCI experienced comparable in-hospital outcomes. In cases where the lesion was heavily calcified and challenging to treat using retrograde CTO-PCI, RA can be a viable option for experienced operators.

### Electronic supplementary material

Below is the link to the electronic supplementary material.

Supplementary Material 1

## Data Availability

The data underlying this article are available in the article and in its online supplementary material.
