# Supplementary material for: Safety and feasibility of rotational atherectomy (RA) versus conventional stenting in patients with chronic total occlusion (CTO) lesions: a systematic review and meta-analysis
Source: BMC Cardiovasc Disord. 2024 Jan 2;24:4. doi: 10.1186/s12872-023-03673-2 (PMC10763069; doi:10.1186/s12872-023-03673-2)
Supplement: Supplementary file 1 — Supplementary Material 1 [file 12872_2023_3673_MOESM1_ESM.docx]

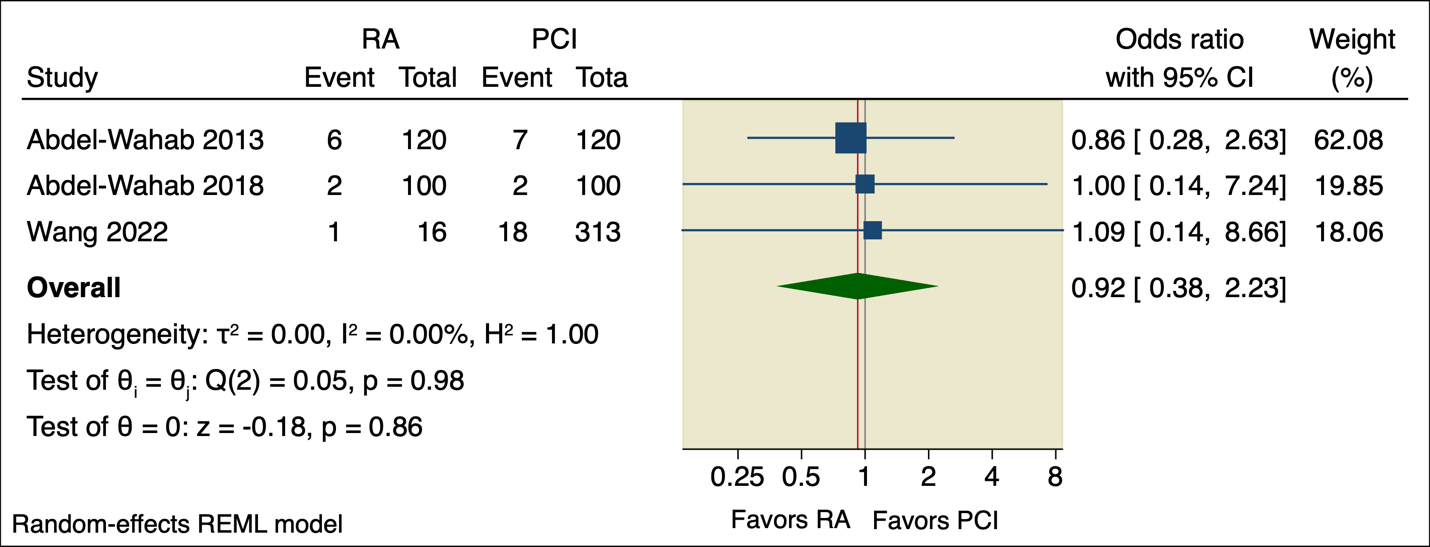


**Supplementary Figure 1:** Sensitivity analysis plot of all-cause death. CI: Confidence interval, RA: rotational atherectomy, PCI: percutaneous coronary intervention.


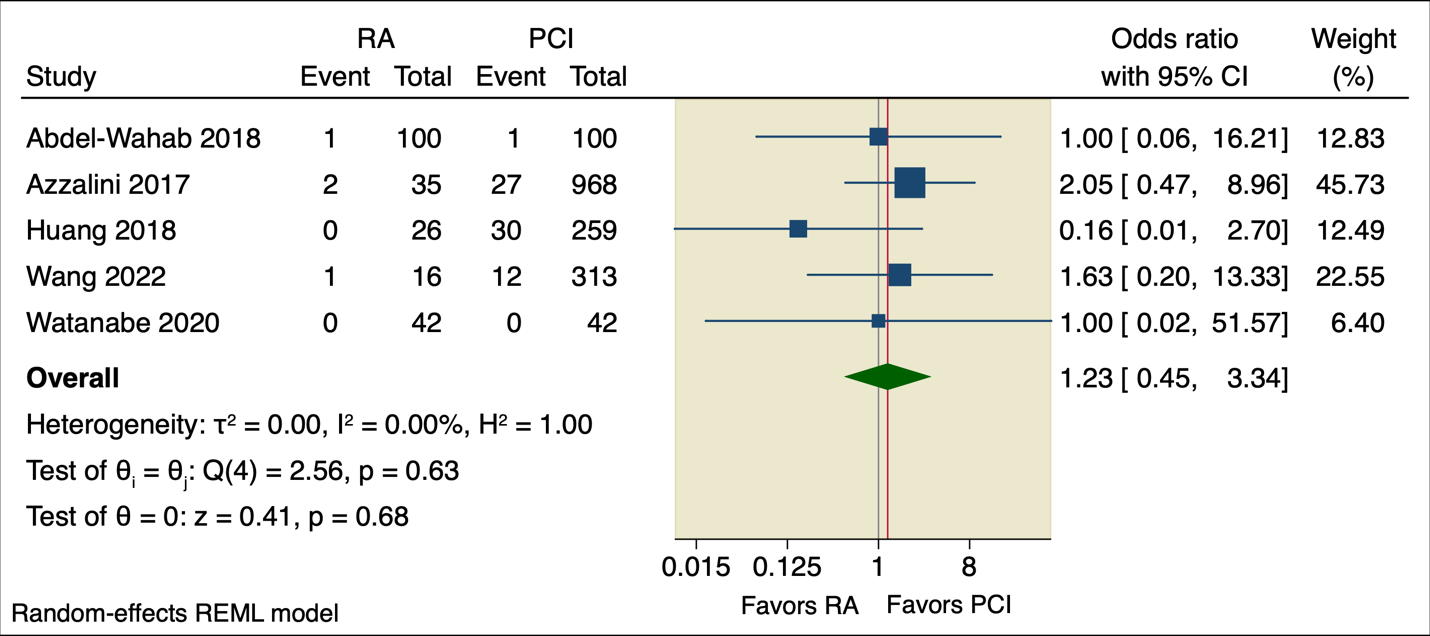


**Supplementary Figure 2:** Sensitivity analysis plot of cardiac death. CI: Confidence interval, RA: rotational atherectomy, PCI: percutaneous coronary intervention.


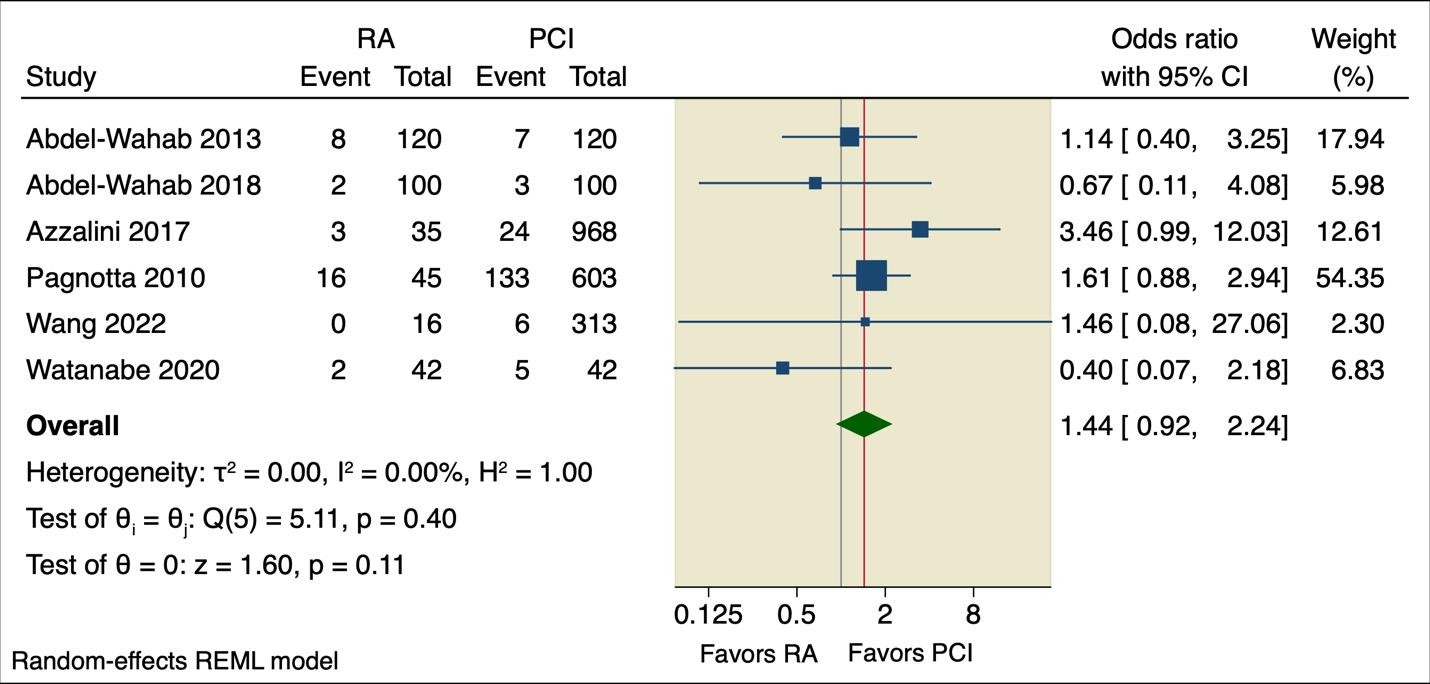


**Supplementary Figure 3:** Sensitivity analysis plot of MI. CI: Confidence interval, RA: rotational atherectomy, PCI: percutaneous coronary intervention.


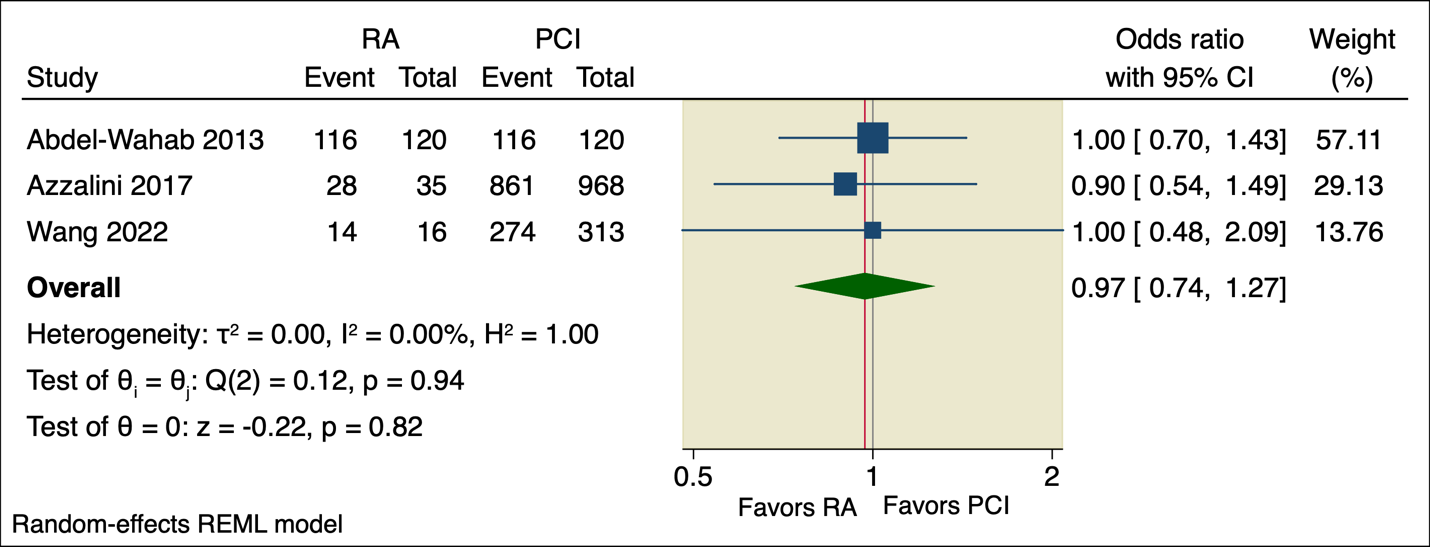


**Supplementary Figure 4:** Forest plot of technical success. CI: Confidence interval, RA: rotational atherectomy, PCI: percutaneous coronary intervention.


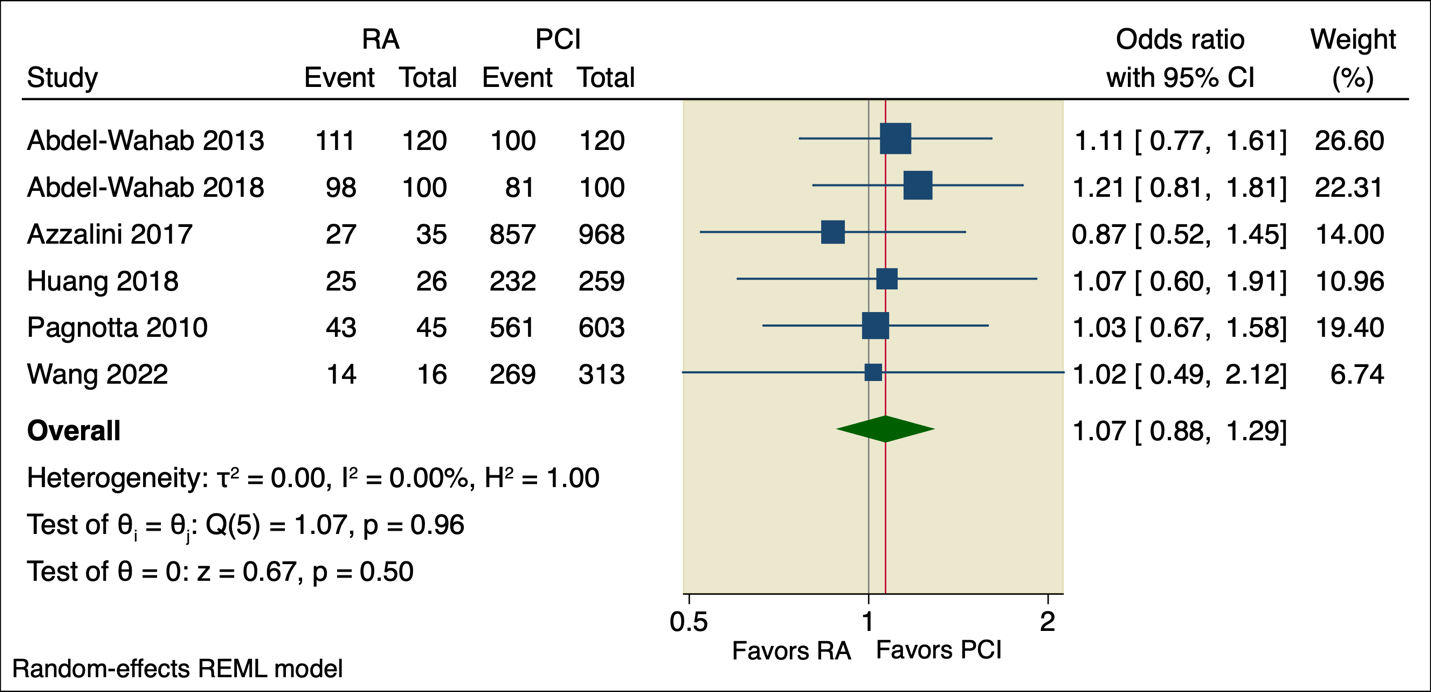


**Supplementary Figure 5:** Forest plot of procedural success. CI: Confidence interval, RA: rotational atherectomy, PCI: percutaneous coronary intervention.


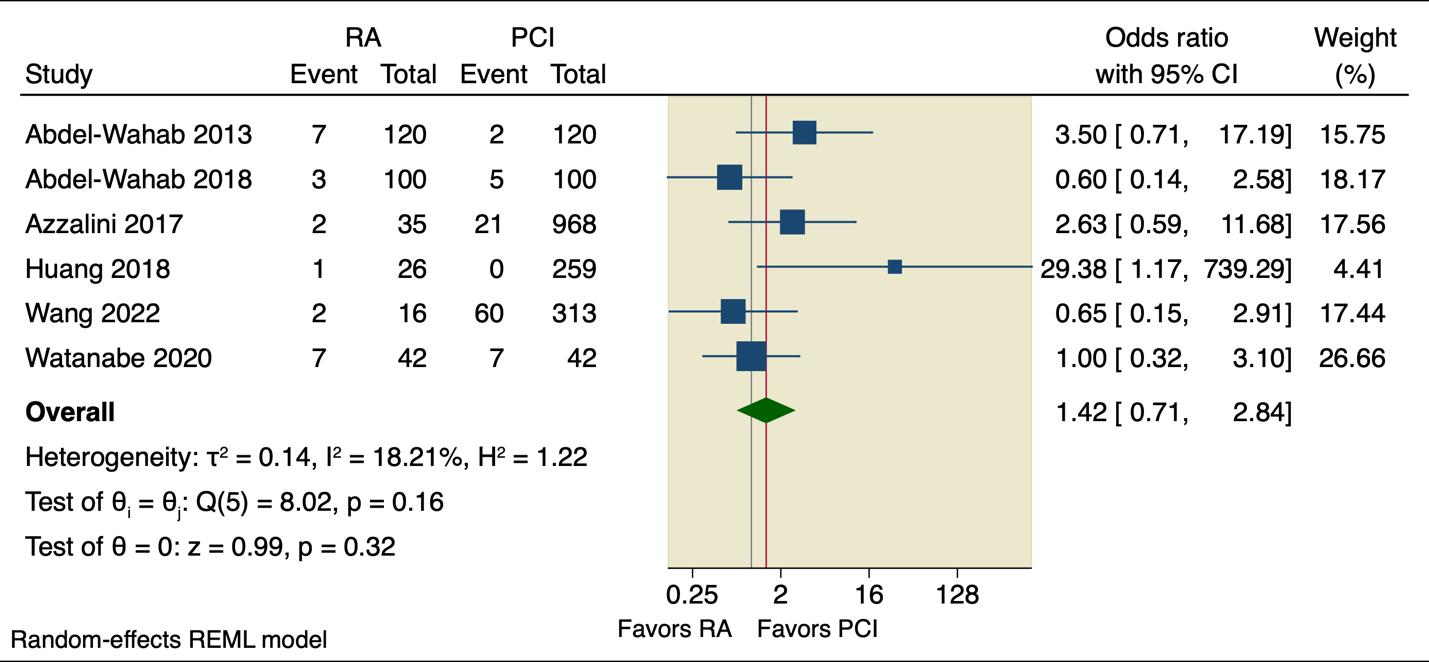


**Supplementary Figure 6:** Forest plot of procedural complications. CI: Confidence interval, RA: rotational atherectomy, PCI: percutaneous coronary intervention.


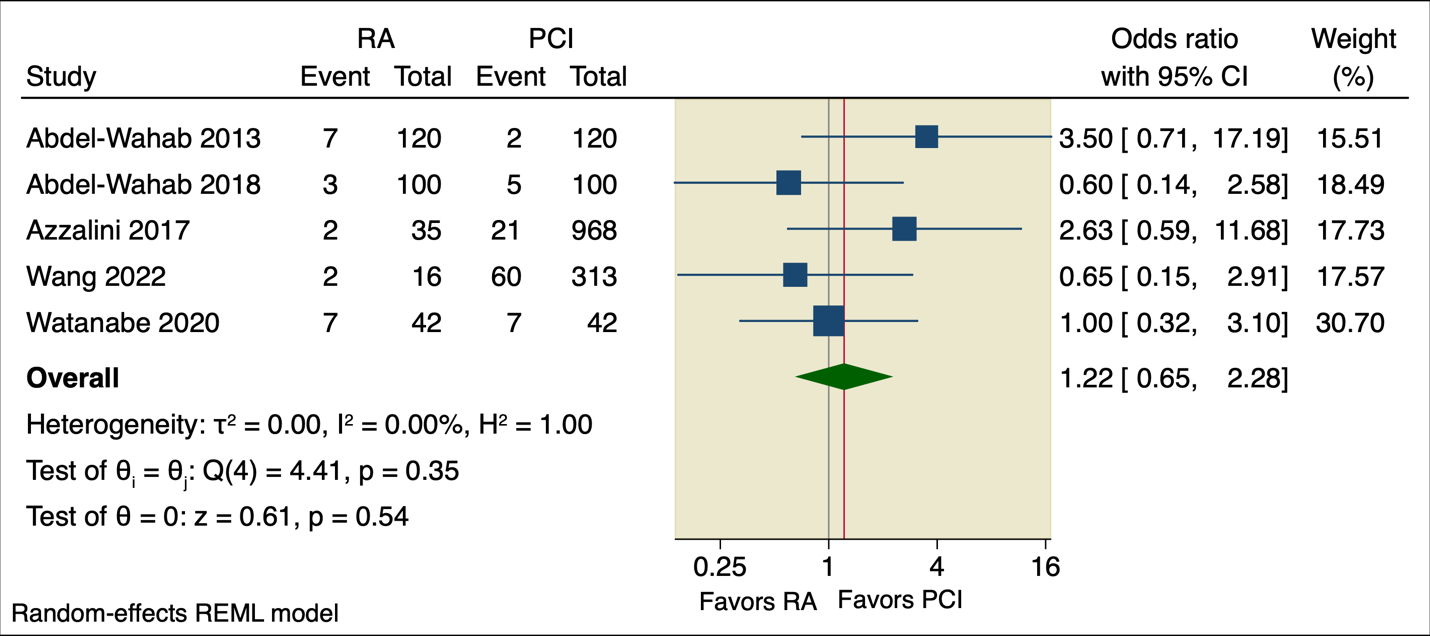


**Supplementary Figure 7:** Sensitivity analysis plot of procedural complications. CI: Confidence interval, RA: rotational atherectomy, PCI: percutaneous coronary intervention.
